# Discovering new perspectives – strengthening autonomy. Students from different healthcare professions interact with patients and provide care in a self-determined and interprofessional manner

**DOI:** 10.3205/zma001560

**Published:** 2022-09-15

**Authors:** Alexandra Wirth, Fabian Berger, Gert Ulrich, Sylvia Kaap-Fröhlich

**Affiliations:** 1 Careum Foundation, Zurich, Switzerland; 2 Careum School of Health, Zurich, Switzerland; 3 Zurich University of Applied Sciences, Bachelor “Biomedical Laboratory Diagnostics”, Wädenswil, Switzerland

**Keywords:** professional health education, patient participation, patient education as a topic, interprofessional education, interprofessional relations, caregivers

## Abstract

The Careum Summer School (CSS) is a learning setting that enables self-regulated learning in an environment in which trainees and students from the various medical, nursing and therapeutic healthcare professions taught in the Swiss education system (upper secondary and tertiary levels A and B) develop project ideas together with patients and their caregivers. The aim of this learning setting is to promote a positive attitude among trainees and students towards interprofessional collaboration that includes patients as cooperation partners.

**Objective:** The evaluation examines the extent to which trainees’ and students’ attitudes towards interprofessional collaboration changed. Information was also obtained on the experiences patients and their caregivers had during their participation in the CSS programme.

**Methodology:** A total of 69 trainees and students were given access to an online survey in the form of the German version of the University of the West of England Interprofessional Questionnaire (UWE-IP) one week before the CSS programme began and six weeks after it concluded. Problem-focused interviews were also conducted with 11 patients and their caregivers.

**Results: **The attitudes of the trainees and students in the UWE-IP Interprofessional Learning Scale improved significantly after the CSS programme was conducted (median t1=22.0/t2=16.0). The effect size was r=0.839 (Wilcoxon test for dependent samples). No significant results could be identified for the other three UWE-IP scales. Patients and their caregivers reported that they were able to actively participate in the CSS programme and felt valued and appreciated.

**Conclusion:** The CSS offered a learning environment in which all participants were able to exchange knowledge and information in an interprofessional manner and work collaboratively on the development of a project idea – for example an interprofessional competency passport with a spider diagram.

## Zusammenfassung

Die Careum Summer School (CSS) ist ein Lernsetting, welches selbstgesteuertes Lernen ermöglicht und in dem Lernende und Studierende aus medizinischen, pflegerischen und therapeutischen Gesundheitsberufen des Schweizer Bildungssystems (Sekundarstufe II, Tertiär A und B) in Zusammenarbeit mit Patient*innen und Angehörigen Projektideen entwickeln. Das Lernsetting hat zum Ziel, die Einstellung der Lernenden und Studierenden gegenüber interprofessioneller Zusammenarbeit unter Einbezug von Patient*innen und Angehörigen zu fördern.

**Zielsetzung: **Die Evaluation prüft, inwiefern sich die Einstellung gegenüber der interprofessionellen Zusammenarbeit bei Lernenden und Studierenden ändert. Zudem wurde erhoben, wie Patient*innen und Angehörige die Teilnahme an der CSS erlebten.

**Methodik:** Neunundsechzig Lernende und Studierende bekamen eine Woche vor und sechs Wochen nach der Durchführung der CSS einen Online-Fragebogen mit der deutschen Version des University of the West of England Interprofessional Questionnaire (UWE-IP). Mit elf Patient*innen und Angehörigen wurden problemzentrierte Interviews geführt.

**Ergebnisse: **Die Einstellung der Lernenden und Studierenden in der UWE-IP Skala „Interprofessionellem Lernen“ verbesserte sich signifikant nach der Durchführung der CSS (Median t1=22.0/t2=16.0). Die Effektstärke liegt bei r=0.839 (Wilcoxon-Test für abhängige Stichprobe). Für die anderen drei UWE-IP Skalen waren keine signifikanten Ergebnisse feststellbar. Patient*innen und Angehörigen berichteten, dass sie sich aktiv einbringen konnten und sich wertgeschätzt fühlten.

**Schlussfolgerung:** Die CSS bietet einen Lernraum, in dem sich alle Beteiligten interprofessionell austauschen konnten und kollaborativ eine gemeinsame Projektidee, wie beispielsweise einen interprofessionellen Kompetenzpass mit Spider Diagramm, entwickeln konnten.

## 1. Introduction

Studies show that interprofessional collaboration has the potential to improve the quality of care in the healthcare system, which would also help reduce healthcare costs. Interprofessional collaboration has also been shown to increase patient satisfaction [1], [2]. In addition, it has been demonstrated that trainees and students can benefit from interprofessional learning settings [3]. However, studies have also shown that the manner in which trainees and students learn is not sufficiently interprofessional in nature, which means they are not properly prepared for interprofessional collaboration after they begin working in the healthcare sector [4]. Patients and their caregivers are now able to increasingly contribute to the medical treatment process by the knowledge they have gained from their own experience (expert by experience [5]; user involvement [6], [https://www.mielen.fi/experts-by-experience/]) in order to help improve the quality of care in the healthcare system and thus increase the satisfaction of patients and their caregivers as well. The University of British Columbia, for example, has developed an Interprofessional Health Mentors Program that allows trainees and students from different health disciplines to work for three semesters with people who have a chronic condition or disability to learn more about the condition and how it affects each person’s life. The trainees and students benefit here from the extensive experience these chronically ill people have with their condition, whereby this also promotes the development of feelings of empathy [7]. In their qualitative study on “Patient involvement in interprofessional education”, Romme et al. [8] stress the fact that the involvement of patients and their caregivers represents a new approach for promoting person-centred and collaborative skills among students, whereby this also supports the approach utilised by the Careum Summer School (CSS). There is still not enough of this type of involvement of patients and their caregivers in the context of training and continuing education programmes. The CSS thus makes a contribution to promoting the inclusion of the knowledge and experience of patients and their caregivers in the education and training of students and trainees, as well as interaction between the two groups.

## 2. Project description

With its two core topics of interprofessionality (IP) and self-determination for patients and their caregivers, the CSS promotes a process of reflection on collaboration between all participants in practice as a means of improving quality of care and thus increasing the satisfaction of patients and their caregivers as well. Here, patients and caregivers contribute their expertise in a self-determined and autonomous manner (participation) and feel as if they have been integrated into the social environment in the context of treatment [9]. To make learning with, from and about each other possible [10], a decision was made to establish a self-regulated learning setting [11]. This means that the learning setting allowed for both social learning and learning that focuses on problem solving. Participants worked in a project-focused manner and developed their own questions, which ultimately led to the development of project ideas. During the CSS programme, the trainees, students, patients and patients’ caregivers assumed responsibility for their own learning process in order to be able to benefit from each other’s knowledge and experience [12]. They were supported throughout the programme by moderators who provided assistance with content and organisational issues when needed. 

The goal of the CSS is thus to promote interprofessional collaboration by providing a self-regulated setting in which programme participants can develop a project idea that is designed to improve the everyday clinical experience. The project ideas that were developed – for example an interprofessional competency passport with a spider diagram, a “Do you need advice or help?” flyer in a paediatric hospital room (to promote the autonomy of minors) and a patient board (with an app) for improving the planning of interprofessional sessions – all have a formative character. Nevertheless, the primary learning objective is to have participants undergo a joint learning process in which they collaborate in an interprofessional setting that also promotes social interaction.

The evaluation examined the extent to which the CSS achieved its goal of improving interprofessional collaboration and mutual understanding between trainees and students from the various health professions and the participating patients and their caregivers.

The following research questions arise as a result:

1. What impact does participation in the CSS programme have on trainees’ and students’ perceptions and attitudes regarding interprofessional learning and interprofessional collaboration?
2. What do the participating patients and their caregivers think about the CSS?

## 3. Methods

The CSS was held from 9–10 July 2019 at the Careum Campus in Zurich. The participating trainees and students were recruited from the practice institutions and universities (see table 1 (Tab. 1)). The participating trainees and students were in the late semesters of their practical training at the time of the summer school, which offered them the opportunity to spend two days working with patients and their caregivers on a topic the patients and caregivers felt was important to them [13]. A total of 69 trainees and students in various stages of their education (upper secondary and tertiary levels A and B) participated, along with 12 patients with chronic conditions, caregivers of patients with chronic conditions and young carers (children, teenagers and young adults who support or care for a person close to them). Participants’ ages ranged from the mid-20s to late 50s. The trainees and students were supported by five moderators from practice institutions.

A mixed methods approach was used to ensure the questions posed by the evaluation could be answered. In view of the low number of participating patients and caregivers in particular, a parallel design was also chosen to facilitate subsequent research. The results were analysed independently of one another. The methods used were an online questionnaire and problem-focused interviews [14]. This study was also presented to the Zurich Cantonal Ethics Commission as part of a request for a Clarification of Responsibility. The Ethics Commission issued a Clarification of Responsibility (BASEC No. Req-2019-00620) and declared that it was not responsible. All participants signed a declaration of consent, and were recruited on a voluntary basis in accordance with international regulations, declarations and guidelines. 

### 3.1. Quantitative survey and analysis – trainees and students 

To determine the extent to which the CSS programme influenced trainees’ and students’ attitudes towards interprofessional collaboration, the trainees and students were surveyed one week before the CSS began (t1) and six weeks after it ended (t2). To this end, Heidelberg University Hospital agreed to allow the study to use the German version of the University of the West of England Interprofessional Questionnaire (UWE-IP) [15]. The questionnaire has four scales:

1. Communication and Teamwork (9 items),
2. Interprofessional Learning (9 items),
3. Interprofessional Interaction (9 items)
4. Interprofessional Relationships (8 items).

The measurements were performed using a four-point (Communication and Teamwork) and five-point Likert scale, respectively. All four scales have cumulative scores, with lower scores indicating a more positive attitude and higher ones indicating a more negative attitude [16], [17], [15]. A link to the survey was sent to the trainees and students via email. Measures were taken to ensure participant pseudonymisation: To identify participants for the follow-up survey, brief questions were used to generate an individual identification key (combination of five letters and numbers) for each person. The identification keys were later used to merge the t1 and t2 data sets.

#### 3.1.1. Description of the participants

A total of 35 of the 69 participating trainees and students filled out the questionnaire for the first online survey (t1). This corresponds to a survey participation rate of 51 per cent. Of the 35 individuals who completed the questionnaire, 28 were women and six were men; one person did not provide any information on their gender. The arithmetic mean for age was 23.94 years, the median was 22. The youngest survey participant was 16 and the oldest was 46. The breakdown in terms of healthcare professions was as follows: the vast majority (19, or 54.3 per cent) of the survey respondents were active in the nursing segment, while the medical and therapeutic segments accounted for eight respondents each. A total of 24 people participated in the second online survey (t2). This corresponds to a survey participation rate of 35 per cent.

Within the dependent sample, it was possible to identify ten individuals who participated in both surveys (t1 and t2). This means that the overall participation rate for the two dependent samples was 14 per cent.

##### 3.1.2. Statistics

The data was descriptively analysed using IBM SPSS v.26. To identify differences in attitudes from before and after the CSS intervention, non-parametric methods (distribution-free tests) were used, whereby this approach was necessitated by the low number of respondents and the fact that the data for the dependent t1 and t2 samples was not normally distributed. The Wilcoxon test was also used for the t1/t2 dependent samples, and the effect size was determined according to Cohen [18]. The UWE-IP questionnaire is divided into four thematic blocks, which means that on the basis of the significance level of p=0.05 and due to the Bonferroni correction, the p-value in the identification of statistically significant differences has to be divided by four (p=0.05/4=0.0125) [19]. 

#### 3.2. Qualitative collection and analysis of the interview material – patients and their caregivers

Due to the low number of cases, and to ensure the openness of the results of the interviews, a qualitative research design was chosen for the patients and their caregivers. Within the framework of this design, the problem-focused interviews [14] allowed to set the reference point on the patients and their caregivers while also framing the discussion context through the use of a guideline. A total of 11 of 12 patients participated in the interviews, which usually took 30 to 40 minutes. Interviews were conducted with three male and four female patients with chronic conditions, and with one male and three female caregivers. The interviews were conducted after the CSS, at which time the declarations of consent to the individual interviews had already been signed and submitted. Among others, the interview guideline included questions about

1. impressions and experiences during the CSS programme,
2. what the discussions in the working groups and the plenary session were like for the interviewees and
3. the contribution the interviewees thought the CSS could make to their ability to cope with their illness – and how the CSS might make it easier for caregivers to provide care.

The interviews were recorded, transcribed and anonymised. The analysis was performed with the help of MaxQDA^®^ software. In an initial step, the categories were derived inductively from the interview material by a researcher (FB). After that, a second researcher (AW) sequentially coded the text material once again and then reviewed it. In line with Mayring and Frenzl [20], the coding processes were assessed in a content analysis in three joint meetings.

## 4. Results

### 4.1. Results of the quantitative survey of trainees and students

**Figure 1:**
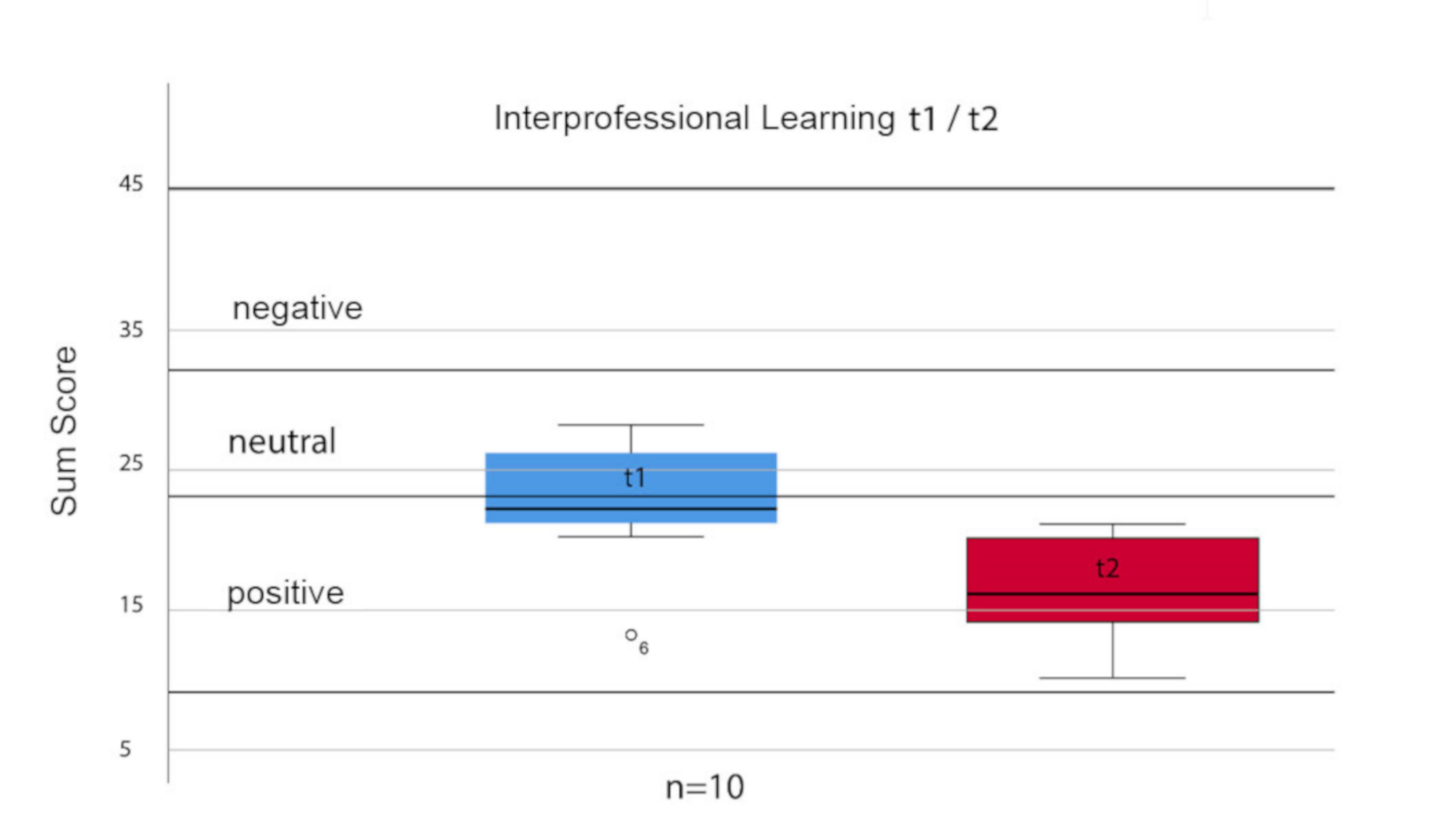
Box plots of the dependent sample t1 vs. t2: UWE-IP – Interprofessional Learning Scale. The assessments (negative, neutral, positive) of the cumulative scores for the UWE-IP – Interprofessional Learning Scale were made according to Pollard et al. [17]

The attitudes of the ten trainees and students as assessed via the UWE-IP Interprofessional Learning Scale improved significantly after the CSS programme was conducted (median t1=22.0/t2=16.0; p=.008). The effect size was r=0.839 and corresponds to a strong effect (see table 2 (Tab. 2) and table 3 (Tab. 3)). No statistical significant differences could be identified for the other three UWE-IP scales (Communication and Teamwork, Interprofessional Interaction and Interprofessional Relationships). Figure 1 (Fig. 1) shows the differences between the two surveys (t1-t2) with regard to the Interprofessional Learning Scale. The assessments of “negative”, “neutral” and “positive” correspond to the proposed analysis method [17], [15], [1].

#### 4.2. Results of the qualitative survey regarding interviews with patients and their caregivers

The qualitative content analysis of the interview material led to the identification of six main categories and 19 subcategories (see table 4 (Tab. 4)). Some of the participating patients and caregivers were surprised by the large number of medical, nursing and therapeutic healthcare professions that exist. Some also didn’t know that many healthcare professionals themselves do not realise how important it is in terms of quality of care that different professions work together in line with the principles of interprofessional collaboration. Once the patients and caregivers understood this, several of them expressed the belief that it’s also important for them to be able to make decisions regarding the type of medical treatment they or their caregivers should undergo. The interview material also revealed that patients and their caregivers were less concerned with learning new things themselves than with sharing their own knowledge and experiences with the trainees and students. The patients and caregivers interviewed described the opportunity to actively participate in the discussion groups and the plenary session, discuss their personal medical histories, and participate in the development of a project idea as a positive experience of social participation. They also said that they did not feel burdened or experience any stress to speak of during their participation in the CSS programme. While some criticism was voiced regarding the topics discussed during the CSS, this tends to indicate a certain ability to actively address these topics rather than a type of dysfunctional burden. One example of such a topic relates to the hierarchy of healthcare professions and the effect it has on interprofessional collaboration. The issue of hierarchy was addressed separately and independently in three different groups and thus played a more important role in the discussions than certain other issues. Patients and their caregivers did report that they felt burdened mostly by the length of the CSS and the intense discussions, although it should be pointed out that they were able to retreat to a quiet room that had been set up for them – and many of them did just that. The patients and their caregivers said they valued the interprofessional learning setting with the trainees and students from the various healthcare professions, which allowed them to develop and present their project ideas with trainees and students in a self-regulated learning environment. Here, the patients and caregivers understood that the setting enabled them to contribute their ideas and experiences. One individual from the patient/caregiver group actually discovered during the CSS that they themselves possessed certain social skills that they had previously been unaware of. This aspect is very much worth mentioning because the individual in question has Asperger syndrome, which is a condition that makes social interaction a very challenging experience. When asked about the organisation of the CSS, patients particularly expressed their satisfaction with the accommodations and the pleasant environment. They were also pleased by the fact that the importance of treating one another with respect was highlighted at the beginning of the CSS, and they reported that such respectful conduct was the norm throughout the programme. However, they did criticise the lack of a specific goal for the CSS, with some patients and caregivers pointing out that the instructions given during the CSS were not sufficiently precise. Several patients/caregivers also expressed a desire for more guidance with regard to defining the topics to be discussed. Many also criticised what they believed was an excessive focus on the topic of “hierarchy”, which made it seem to them that the topics they were interested in were somehow viewed as less important.

## 5. Discussion

The evaluation of CSS 2019 shows that trainees, students, patients and their caregivers were all able to benefit in their own way from the programme. In the case of trainees and students, the acceptance of interprofessional learning formats increased, which accordingly had a positive effect on the assessments for the UWE-IP – Interprofessional Learning Scale. The practice institutions and universities from which the trainees and students were recruited are also aware of this, and they therefore welcome the use of interprofessional learning settings for their trainees and students. The evaluations of the results were discussed with the practice institutions and universities in a separate workshop [21]. Due to the low number of participants in the online survey, as well as the Bonferroni correction, our study did not allow us to make any statistically significant statements about the other three UWE-IP scales (Communication and Teamwork, Interprofessional Interaction and Interprofessional Relationships). In addition, no statements could be made regarding the attitudes of subgroups (gender, individual healthcare professions). Mink et al. [22] were also unable to identify any statistical significant differences between genders and healthcare professions in the UWE-IP. In this connection, it should also be pointed out that the associated study, which ran for a longer period (three months), was unable to show any statistical significant effects. This fact needs to be considered when interpreting our results, as the follow-up survey of trainees and students occurred at a much earlier point in time (six weeks after CSS 2019 ended). Mink et al. pointed out the ceiling effect (overestimation of the positive manifestations in a scale), as well as the positive expectations of the students, who volunteered to participate in the intervention. We agree with the associated conclusions, since given the low number of cases (n=10) in particular, it can be assumed that only highly motivated trainees and students participated in both surveys, meaning that attitudes towards interprofessional learning were overestimated when the surveys were taken, and in particular after the intervention. Other studies show that trainees and students benefit in particular in connection with the Communication and Teamwork Scale and the Interprofessional Learning Scale [23], while results in connection with the Interprofessional Interaction Scale can become more negative after interventions because when people from different healthcare professions come together, it can actually lead to a reinforcement of preconceptions about the other disciplines in each case [24].

The involvement of patients and their caregivers in CSS 2019 clearly shows the extent to which this group was surprised by the lack of knowledge people from each profession had about the others. According to the statements made by patients and caregivers, this had a positive effect in that it reinforced their own attitudes about the importance of taking on more responsibility for themselves and their caregivers with regard to their own or their caregivers’ medical treatment. 

The issue of hierarchy was addressed separately and independently in three different groups and thus played a more important role in the discussions than certain other issues. This in turn was a result of the self-regulated learning approach. There was a drawback here, however, in that interprofessional collaboration with patients and their caregivers was restricted somewhat by the specific focus on the hierarchy of healthcare professions. One condition for a future CSS could be that the interprofessional groups with patients and caregivers should no longer be broken up after a topic is selected, which would lead to more guidance for the groups.

The fact that the evaluation selected for the CSS was able to show that attitudes towards interprofessional learning improved should be stressed here. In addition, the patients and caregivers felt that the CSS added something positive in terms of their social skills and social environment. Many patients and caregivers were particularly surprised and impressed by the new knowledge they gained regarding the number of healthcare professions in the healthcare system. However, no other significant changes could be identified in the other UWE-IP scales. Compared to Pollard et al. [17], the intervention here was too short, meaning that a two-day CSS is likely to have less of an effect, while on the other hand the Bonferroni correction leads to an even lower significance level. It would have been better to have had a higher number of cases for the associated samples. In this regard, it makes sense to examine the option of making participation in the surveys mandatory – i.e. as part of a programme evaluation. The time period is another factor, as the surveys and interviews were conducted during the summer holiday period. In addition, the pre-test / post-test design selected (two surveys at two different times) means that it is not possible to determine whether the changes in attitude that were observed will be maintained over a longer period. Finally, the evidence of a positive effect brought about by the CSS as regards the acceptance of interprofessional courses is limited since a non-experimental evaluation design was selected. 

## 6. Conclusion

Suitable formats for exchange and interaction need to be made available if patients and their caregivers are to be incorporated into the interprofessional environment. This means that trainees and students not only have to be aware of everything going on in their interprofessional environment; they also need to understand that patients and their caregivers become experts by experience [5] as a result of their ongoing chronic conditions (user involvement [6]) [https://mielen.fi/experts-by-experience-program/]). In other words, patients and caregivers acquire specialised knowledge and experience that has a major impact on the way they deal with themselves, their illness and the healthcare system. This knowledge and experience make it possible for trainees and students on the one hand, and patients and their caregivers on the other, to meet on equal terms, and it also ensures that information is communicated in a targeted manner to those who need it. In terms of practical application, this means that appropriate learning settings need to be made available by the relevant institutions so that future health professionals can experience an interprofessional exchange as they work with each other and with patients and their caregivers – in line with the principles of “organisational learning” [25]. The exchanges and interaction can thus serve as a foundation for the further development of, and reflection on, relationships with other health professionals from different disciplines, as well as with patients and their caregivers. It is also important that patients and their caregivers are empowered and develop a high degree of health literacy [26], as well as a feeling of self-determination and autonomy, so that they realise that they too can and should be able to make decisions. The format used for the CSS will be further developed and a new CSS will be conducted again in 2022 in cooperation with older people and experts from the healthcare and social service sectors. 

## Previous presentations

Previous presentations [27], [28].

## First authorship

Alexandra Wirth and Fabian Berger share the first authorship.

## Acknowledgements

The authors would like to thank all those who participated in the study. We understand that the time and resources available to all participants are limited and very valuable. Their stories, and the experiences shared in our interviews, have had a major impact on our research programme. 

Finally, last and most definitely not least, we would like to thank both Dr. Katherine Pollard from the University of the West of England in Bristol (UWE Bristol), as well as the Department of General Practice and Health Services Research at Heidelberg University Hospital in Germany, which translated the questionnaire, for making it possible for us to use the UWE IP questionnaire. 

## Competing interests

The authors declare that they have no competing interests. 

## Figures and Tables

**Table 1:**
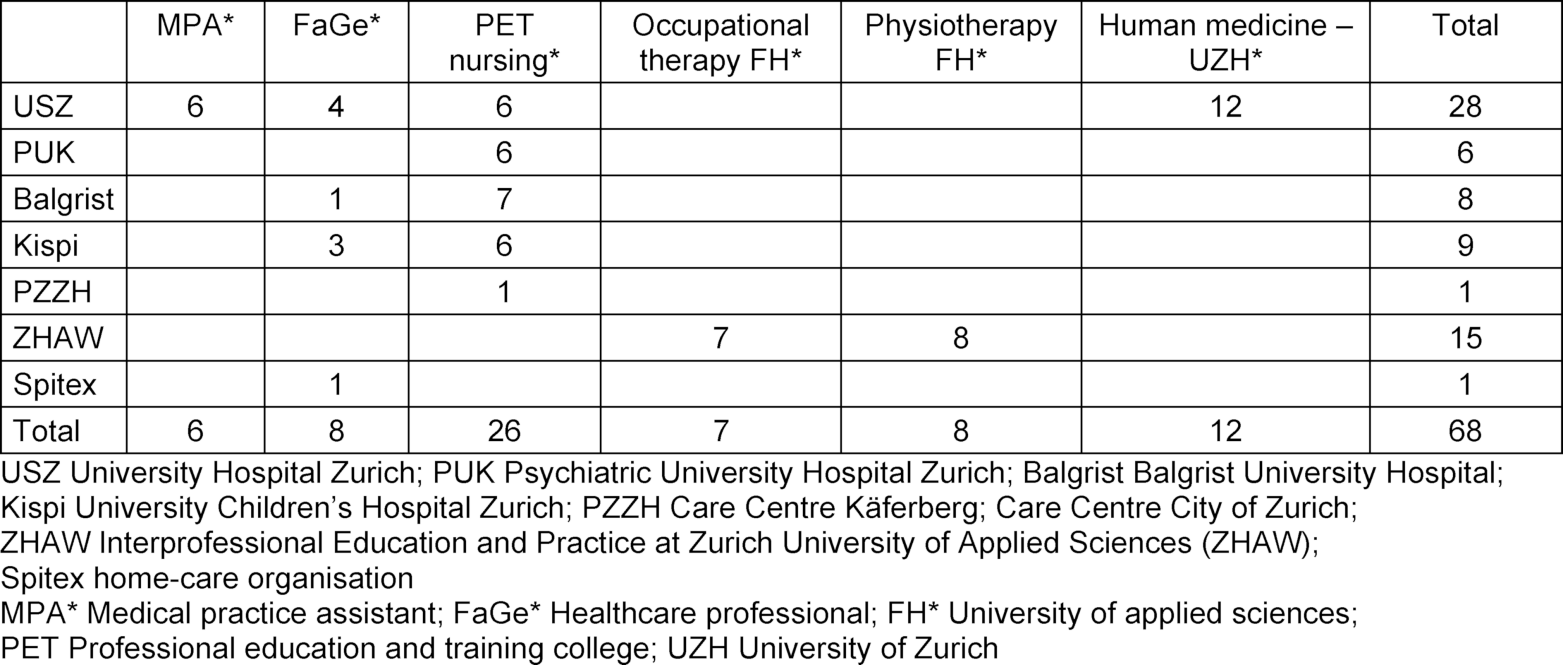
Trainees and students from various practice institutions and healthcare fields

**Table 2:**
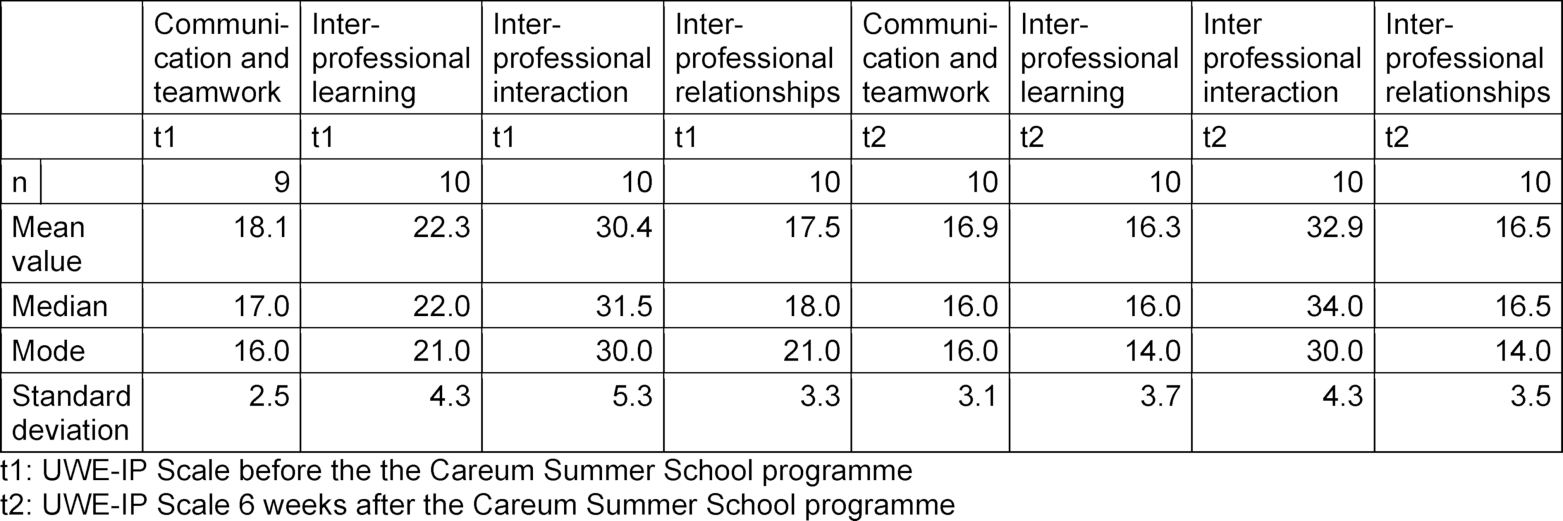
Dependent samples between t1 and t2: Trainees and students

**Table 3:**
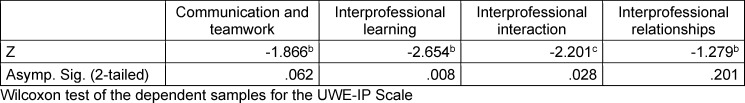
Central tendency of the UWE-IP scalesbefore and after the Careum Summer School programme

**Table 4:**
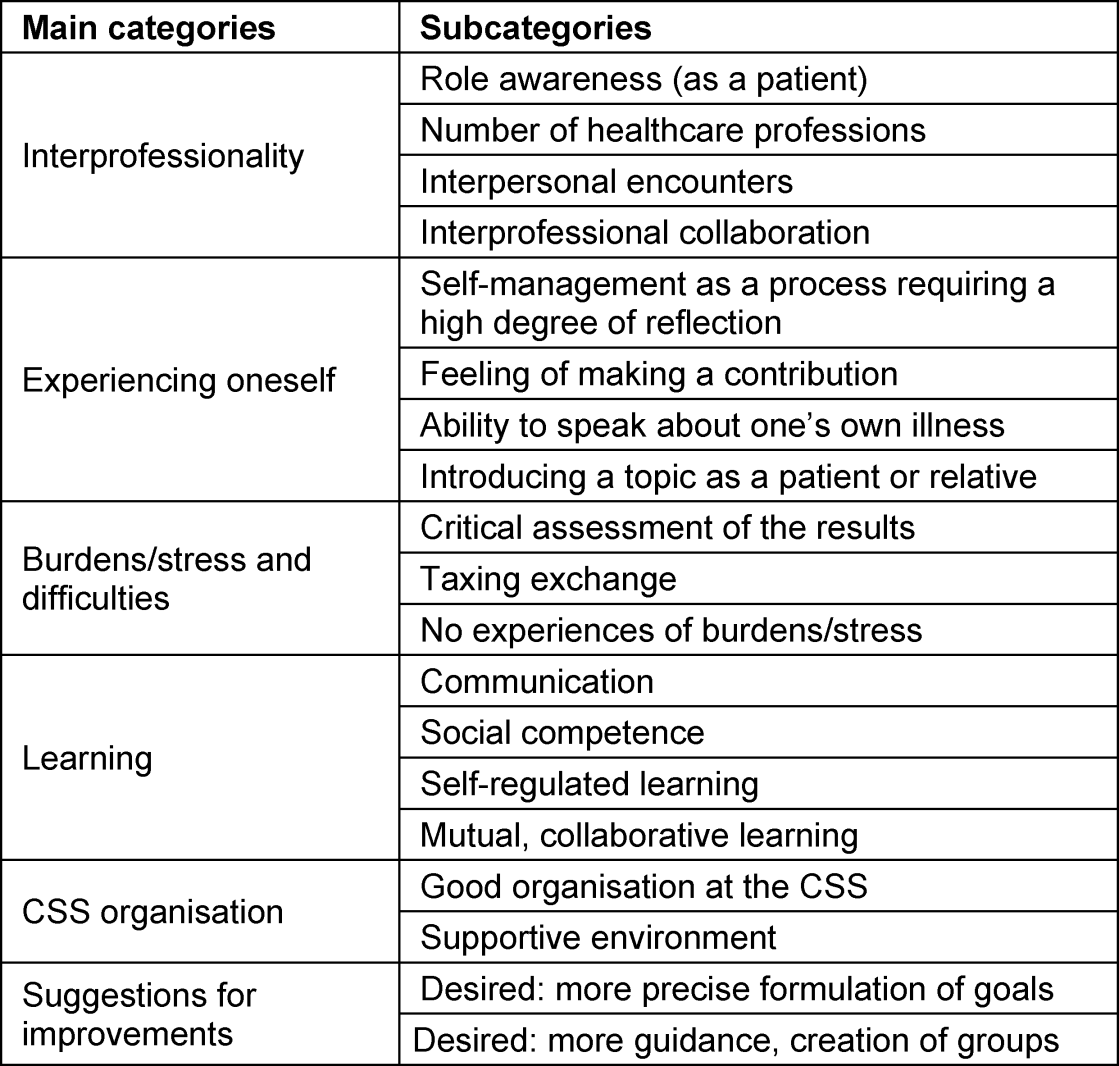
Main categories and subcategories of the questions for the interviews with patients and relatives
